# Immediate effects of the respiratory stimulation on ventilation parameters in ischemic stroke survivors: A randomized interventional study (CONSORT)

**DOI:** 10.1097/MD.0000000000017128

**Published:** 2019-09-20

**Authors:** Lucyna Ptaszkowska, Kuba Ptaszkowski, Tomasz Halski, Jakub Taradaj, Robert Dymarek, Malgorzata Paprocka-Borowicz

**Affiliations:** a Department of Physiotherapy, Opole Medical School, Opole; b Department of Clinical Biomechanics and Physiotherapy in Motor System Disorders, Faculty of Health Science, Wroclaw Medical University, Wroclaw; c Department of Physiotherapy, Opole Medical School, Opole; d Institute of Physiotherapy and Health Sciences, Academy of Physical Education in Katowice, Katowice, Poland; e College of Rehabilitation Sciences, University of Manitoba,Winnipeg, Canada; f Department of Nervous System Diseases, Faculty of Health Science, Wroclaw Medical University; g Department of Clinical Biomechanics and Physiotherapy in Motor System Disorders, Faculty of Health Science, Wroclaw Medical University, Wroclaw, Poland.

**Keywords:** ischemic cerebral stroke, proprioceptive neuromuscular facilitation, respiratory parameters

## Abstract

**Background:**

Post-stroke brain damage, which affects the central control of respiration, leads to various respiratory disorders. They can be caused by the weakening of the respiratory muscles and chest movements, which can indirectly contribute to an impairment of the ventilation function. The aim of the study is an objective assessment of the effect of a single-session intervention of respiratory stimulation through Proprioceptive Neuromuscular Facilitation (PNF) on chosen respiratory parameters and the following comparison of these changes with a group in which positioning was used (intra- and intergroup comparison).

**Methods:**

This was a randomized interventional study evaluating the respiratory parameters depending on the applied respiratory stimulation in patients after ischemic stroke. The patients qualified to participate in the study were randomly assigned to 1 of 2 groups: PNF-treated group – in which respiratory stimulation through PNF was used, PNF untreated group – in which positioning was used. The research procedure consisted of several stages. First, an interview was conducted with each participant and basic data was collected. Then, spirometry was conducted, after which each patient underwent a single-session intervention according to their assigned group. Finally, the patients were given another spirometry examination. The main outcomes will be to compare the results of a spirometry test (FVC, FEV1, FEV1/ FVC%, PEF) before and after single-session intervention and between groups.

**Results:**

Based on the inclusion and exclusion criteria for the study, 60 patients took part in the measurement. The values of FEV1/FVC% were higher in PNF-treated group than in PNF-untreated group, if the post-intervention measures (*P* = .04) are considered. The difference between the pre- and post-intervention results of the FEV1/FVC% values in PNF-untreated group was substantially lower than in PNF-treated group (*P* = .001).

**Conclusion:**

A single application of respiratory stimulation through PNF positively affect air flow in the respiratory tract. Application of PNF stimulation contributed to an increase in the FEV1/FVC% parameter. However, no positive changes were noted in the other parameters, which would provide proof of the beneficial effect of facilitation on the respiratory system function.

## 1 Introduction

Stroke is one of the most common cardiovascular diseases in the world. It is the main cause of death and permanent disability in adults and one of the main public health problems.^[1,2]^ According to World Health Organization, every year, 15 million people worldwide have a stroke, one-third of which becomes disabled.^[3]^ In Poland, there are 60 to 70 new cases annually. European demographic projections show that the incidence of stroke will increase.^[4]^ It is estimated that between the years 2005 and 2025, the percentage of stroke incidence will rise by 37% in men and 38% in women.^[4]^ The stroke will occur in 35% of the population aged 65 and over, which will grow proportionally in all countries due to demographic changes.^[5]^ This trend will most probably lead to a growing demand for rehabilitation programs directed toward improving patients’ functional status and quality of life.

Focal brain damage symptoms, particularly those concerning neuromuscular coordination, can adversely affect activation of abdominal and chest muscles. It has been noted in the literature that, in stroke survivors, little attention has been paid to the assessment of the muscle function in the trunk.^[6]^ Those patients show the impaired function of the diaphragm and the respiratory muscles, especially on the paretic side of the body, which can lead to asymmetric respiratory chest movements, changes in the respiratory mechanism, and in consequence, to a decreased efficiency of lung ventilation.^[7–12]^

During the evaluation of stroke patients, long-term survivors in particular, too little attention is paid to the functioning of the respiratory system and the analysis of the mechanisms responsible for the breathing process.^[9,11,13–18]^ In central nervous system diseases, respiratory functions usually show restrictive defects, which are caused mainly by the changes in the chest wall or the abdomen.^[16]^

In the early stages of the post-stroke period, anomalies in the respiratory system are not noticeable; however, with time, in cases of major muscle weakness, a significant decrease in the values of vital capacity (VC) and forced vital capacity (FVC) can be observed.^[14–16]^ A decline in the FVC, Forced Expiratory Volume (FEV1), and Peak Expiratory Flow (PEF) parameters occur in stroke survivors regardless of the side of paralysis or paresis^[6,7,11]^ many researchers;^[6,7,10,18]^ attribute the impairment of the lung function to the weakening of the respiratory muscles. Khedr et al^[7]^ noted that the values of FEV1, FVC, and PEF in patients with focal brain damage were almost 2 times lower than in healthy people. Similar results for FVC, FEV1, and FEV1/FVC ratio, (FEV1/FVC%) can be seen in Voyvoda.^[10]^

Post-stroke brain damage, which affects the central control of respiration, leads to various respiratory disorders.^[11]^ They can be caused by the weakening of the respiratory muscles and chest movements, loss of muscular synergy within those structures, and anomalies in the postural control of the trunk, which can indirectly contribute to an impairment of the ventilation function, a decline in the respiratory parameters, and an increased risk of hypoxia.^[1,9,10]^

The null hypothesis was a single-session intervention respiratory stimulation through Proprioceptive Neuromuscular Facilitation (PNF) causes any improvement of the respiratory parameters. I was assumed that PNF improves air flow in the respiratory tract. The aim of the study is an objective assessment of the effect of a single-session intervention of respiratory stimulation through PNF on chosen respiratory parameters and the following comparison of these changes with a group in which positioning was used (intra- and intergroup comparison). The main outcomes will be to compare the results of a spirometry test (FVC, FEV1, FEV1/FVC%, PEF) before and after single-session intervention and between groups

## 2 Methods

### 2.1 Study design

This was a randomized interventional study evaluating the respiratory parameters depending on the applied respiratory stimulation in patients after ischemic stroke. The project was designed by the current guidelines of the CONSORT 2010 statement (CONSORT – CONsolidated Standards of Reporting Trials) for reports presenting randomized clinical trials. Moreover, the study was registered with the international clinical trials platform: Australian and New Zealand Clinical Trials Registry, (no. ACTRN: 12613001315707).

### 2.2 Study patients

The research was carried out in the External Department for Neurological Rehabilitation of the Regional Specialist Hospital in Wroclaw. The study involved ischemic stroke survivors at least 6 months post episode admitted to a neurological rehabilitation ward. All patients had TACI type of stroke with MCA occlusion. The inclusion criteria were as follows: ischemic stroke (at least 6 months post episode), hemiparesis, consent of the patient to participate in the study, permission of the attending physician, the general state of well-being of the patient on the day of examination. The exclusion criteria: sensory aphasia, history of myocardial infarction, chronic respiratory diseases (bronchial asthma, COPD), infection, other respiratory diseases or diseases affecting the work of respiratory muscles (for example ALS), obesity (BMI > 28), past thoracic or abdominal surgery, the Barthel Index <60, lack of consent.

### 2.3 The ethical aspect of the study

The research project was approved by the Bioethics Commission of the Wroclaw Medical University (approval no. KB - 867/2012). All the participants of the project were informed of the purpose of the study and the procedures to be conducted, and they expressed their written consent for participation and processing of their data.

### 2.4 Study protocol and outcomes

The patients qualified to participate in the study were randomly assigned to 1 of 2 groups: PNF-treated group – in which respiratory stimulation through PNF was used, PNF-untreated group – in which positioning was used.

The research procedure consisted of several stages. First, an interview was conducted with each participant, during which the patient was informed about the course of the study, and basic data was collected, such as the patient's age, weight, height, smoking habits, stroke episode, and paretic side. The following stage involved an assessment of the patient's basic daily life activities with the international Barthel scale. Then, spirometry was conducted, after which each patient underwent a single-session intervention according to their assigned group. Finally, the patients were given another spirometry examination.

The spirometry was conducted with the Spirobank USB Spirometer (MIR Medical International Research USA, Inc.), the Winspiro Express software, and the compatible FlowMir disposable turbines with a mouthpiece (MIR Medical International Research USA, Inc.), all of which comply with the ATS/ERS standards. The FlowMir disposable turbines with mouthpiece used to measure volume and flow are digital, which guarantees the accuracy and reproducibility of the results without the need for periodic calibration. Moreover, each turbine is individually packaged, providing maximum hygiene to the patient. The Spirobank USB Spirometer is certified as compliant with the Class IIa requirements of the Medical Devices Directive 93/42/EEC.

The spirometry test was conducted with the patients in a sitting position. Before the test, the patients were informed about the method and procedure. Then, the researcher applied a clip to the patient's nose, which resulted in the patients being able to breathe only through their mouths. The patients were then instructed to take the maximal amount of air into their lungs, tightly seal the mouthpiece with their lips, and exhale as fast as they could for 6 seconds. After that time, the expiratory phase ended with a sound signal produced by the device.^[19–21]^ The whole procedure was repeated three times. The spirometry test was conducted before and 10 minutes after the intervention. The present study compared changes in the following spirometry parameters:

(1) Forced Vital Capacity (FVC, unit: liter [L]) is the volume of air that can forcibly be blown out after full inspiration;
(2) Forced Expiratory Volume in the 1st second (FEV1; unit: liter [L]) is the volume of air that can forcibly be blown out in first 1 second, after full inspiration;
(3) FEV1/FVC is the ratio of FEV1 to FVC (unit: percent [%]);
(4) Peak Expiratory Flow (PEF; unit: liter/second [L/S] is the maximal flow achieved during the maximally forced expiration initiated at full inspiration.

### 2.5 Intervention

After taking spirometry measurements, each patient had the single-session intervention, depending on the allocation to the group. The purpose of the single-session interventions used in the study was to intensify the respiratory movements of the chest and to normalize the muscle tension within the muscles involved in the ventilation process on the affected and healthy side of the body. In PNF-treated group, the intervention consisted of performing respiratory stimulation using the PNF techniques.^[21–24]^ The stimulation was divided into 2 stages:

Stage I: consisted of respiratory stimulation performed on the diaphragm – stimulation of the diaphragm was carried out in the supine position with knees slightly flexed and upper limbs resting on special rollers and wedges. The therapist placed his/her hands on the upper abdominal area of the subject. Application of the stretching stimulus at the final stage of expiration aims to influence the sternal and costal origin of the diaphragm and to activate them for a stronger contraction (i.e., to initiate the inspiration). During the inspiration, the therapist's hands, placed in the same manner, exert a slight pressure/resistance on the raising abdominal integuments (without preventing movement). In this way, the resistance has a facilitating (stimulating) effect on motion. Such respiratory stimulation was performed 3 times. A 1-minute break was used between every single stimulation.

Stage II: consisted of respiratory stimulation carried out on the costal inferolateral regions of the chest (lower costal respiratory pattern). Stimulation of the inferolateral region was performed in the same position as the stimulation at stage I. For this stimulation the therapist placed his hands on the inferolateral regions of the chest, with fingers along the line of the ribs. During the final phase of expiration, the therapist applied pressure (stretching) in the caudal and centripetal direction, at the same time instructing the subject to take a deep breath. Similarly, to the diaphragm stimulation, during inspiration, the therapist exerted a slight resistance on the expanding ribs, without preventing the movement. Stimulation of the costal inferolateral regions was performed three times, with a 1-minute break between each stimulation. The intervention (stage I and II) in the PNF-treated group lasted about 10 to 15 minutes. To avoid the hyperventilation of the subject, there was a 3-minute break between stimulation I and stimulation II.

**Figure 1:**
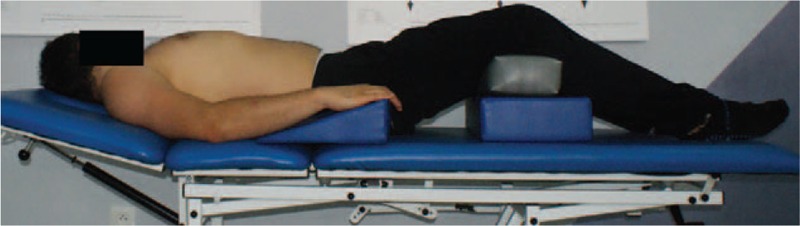
Position of participants in the PNF-untreated group. PNF = proprioceptive neuromuscular facilitation.

In PNF-untreated group, only the patient positioning was used. The patient was lying on his back. Under the lower limbs wedge bolster was placed (Fig. 1). In this position, the diaphragm pathway of breathing is activated, and also, the diaphragm is in an optimally high position, providing a greater inspiratory volume. Flexing the lower limbs results in relaxation of the abdominal integuments and improves the inspiratory effectiveness. Patients remained in this position for 10 minutes.

### 2.6 Statistical analysis and sample size

Statistical analysis was performed using the Statistica 13 (TIBCO Software, USA) software. The sample size was assessed on the bases of pilot studies. These results have not been published so far. The study compared the differences in FEV1/FVC between 2 groups. Prior data indicated that the difference of FEV1/FVC between groups is normally distributed with a standard deviation of 8.5%. If the true difference in the mean of FEV1/FVC between groups is 7.1% than to be able to reject the null hypothesis, 30 subjects will be needed in each group. Power analysis was set at 0.8. The Type I error probability associated with this test of this null hypothesis was 0.05. Arithmetic means standard deviations and the variability range (extreme values) were calculated for measurable variables. For qualitative variables, the frequencies of their occurrence were calculated (percent). All the studied quantitative variables were analyzed with the Shapiro-Wilk test to determine the type of distribution. Differences between the groups were established using the parametric *t* test for independent trials or the nonparametric Mann–Whitney *U* test, depending on the fulfillment of the test assumptions. The comparison of results before and after the intervention was carried out using a *t* test or Wilcoxon matched-pairs test. For all the comparisons the adopted significance level was α = 0.05.

## 3 Results

**Figure 2:**
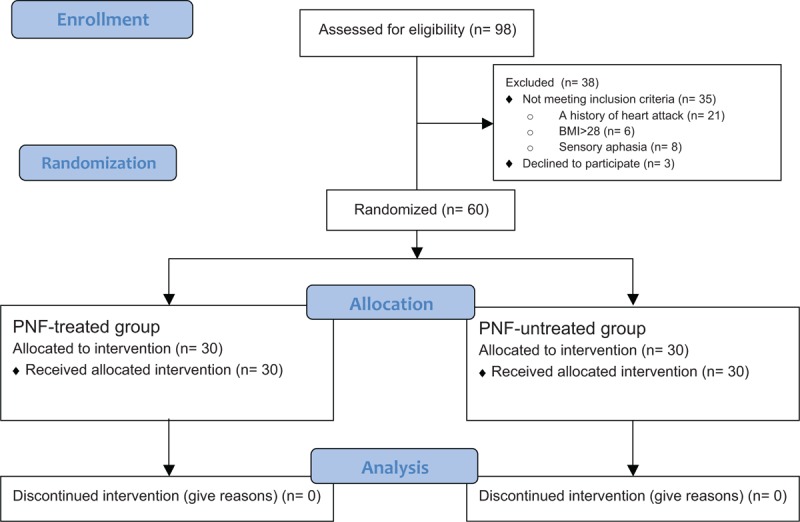
Flowchart of participants (CONSORT flow diagram).

Ninety-eight patients were qualified for the study. Based on the inclusion and exclusion criteria for the study, 60 patients took part in the measurements. Figure 2 presents the flow of patients at each stage of the project.

The detailed characteristics of groups, taking into account age, body mass and height, body mass index (BMI), Index Barthel, affected side, gender, profession, and cigarettes smoking, is presented in Table 1. Both groups were homogeneous in terms of the studied parameters (*P* > .05).

**Table 1:**
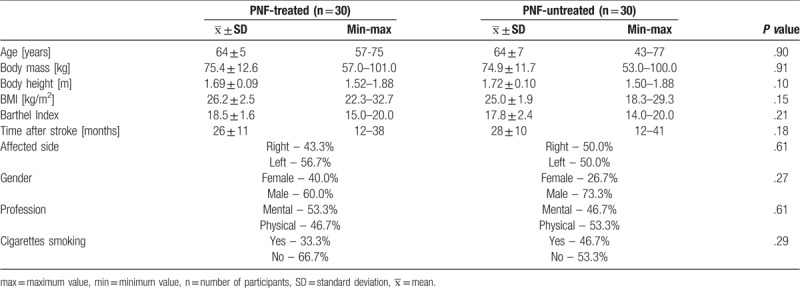
Characteristics of the study groups.

### 3.1 Comparative analysis of the spirometry test results

Table 2 shows the intergroup and intragroup analysis of the spirometry test results (FVC, FEV1, FEV1/FVC%, PEF), including the comparison between the pre- and post-intervention results as well as the difference between those values (delta Δ).

**Table 2:**
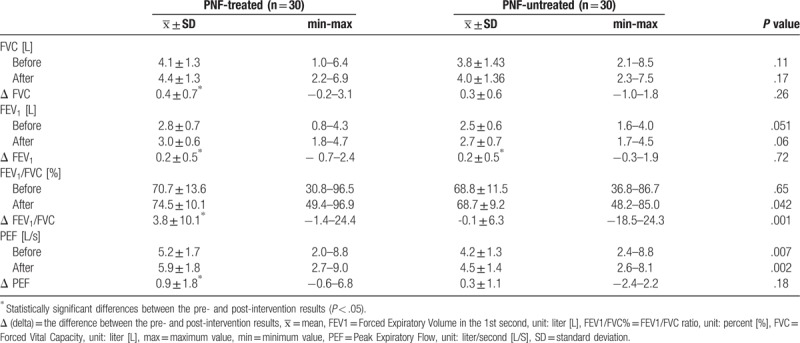
The intergroup and intragroup analysis of the spirometry test results.

The values of FEV1/FVC% were higher in PNF-treated group than in PNF-untreated group, if the post-intervention measures (*P* = .04) are considered. The delta (Δ) of the FEV1/FVC% values in PNF-untreated group was substantially lower than in PNF-treated group (*P* = .001). The rest of the values were statistically insignificant (Table 2).

The intragroup comparative analysis of the FVC and FEV1 parameters showed a statistically significant increase in the value of both FCV (*P* < .001) and FEV1 (*P* = .003) in PNF-treated group. In PNF-untreated group, a substantial rise in value was noted only for FEV1 (*P* = .02). The changes in the value of FVC in the group with positioning were statistically insignificant (Table 2). The analysis of the FEV1/FVC% parameter showed a statistically significant increase in its value only in PNF-treated group (*P* = .002). No statistically significant changes were noted in PNF-untreated group. The intragroup analysis of the PEF parameter showed a substantial increase in its value post-intervention in PNF-treated group. Higher values of PEF in PNF-untreated group were not statistically significant (Table 2).

## 4 Discussion

The main aim of this study was an objective assessment of the influence of the single-session of respiratory stimulation through neuromuscular facilitation on the respiratory parameters in long-term ischemic stroke survivors. The intergroup comparative analysis of the spirometry test results showed that only stimulation through PNF led to a significant increase in the FEV1/FVC% parameter. The analysis of the results pre- and post-intervention showed that respiratory stimulation contributed to an improvement of the FVC, FEV1, and PEF parameters. In a study on the effectiveness of PNF techniques in improving the functioning of the respiratory system in stroke survivors, works by Chang^[25]^ and Song^[26]^ must be mentioned. Chang et al^[25]^ state that a single, 20-minute stimulation through neuromuscular facilitation does not lead to improved values of the VC, FVC, and FEV1 parameters, which is inconsistent with the results of the spirometry tests conducted in this study. The researchers suggest that the lack of improvement might stem from the fact that the described technique has no immediate effect on the respiratory functions. However, it seems that the difference in the outcomes of the interventions might have been caused by the type of positioning used in the described breathing exercise. Unlike the author of this study, who used the lying-on-back position, Chang^[25]^ team applied stimulation while the patients were lying on the side. However, in that position, only the paretic side is affected, which prevents the full expansion of the lung base and makes the full range of motion in the diaphragm function impossible. Song's team^[26]^ used the same starting position to stimulate long-term stroke survivors. Nevertheless, in that case, the analogous respiratory therapy was administered for 30 minutes daily, 5 times a week, for 8 weeks. After that time, the researchers noted a significant increase in the FVC and FEV1 values.

An interesting aspect of both studies is the attempt to assess the relationship between the improvement of respiratory functions and exercise tolerance or trunk control. Aside from the spirometry evaluation of the effect of the stimulation, Chang^[25]^ used the 6 Minute Walk Test (6MWT), and Song^[26]^ performed trunk control assessment using the Trunk Impairment Scale (TIS). In patients subjected to neuromuscular facilitation in the chest area, an improvement was noted both in the distance covered during the 6MWT and in the results of the trunk control assessment. Both Chang^[25]^ and Song^[26]^ point out that positive changes in physical efficiency and body stability can result from the role of respiratory muscles in postural control. Noh et al^[27]^ state that the diaphragm, which is the largest respiratory muscle, aside from permitting proper respiratory function, contributes to postural control, and that its dysfunctions are significant in the pathogenesis of back pain.

The correlation between postural control, spirometry, and strength of the respiratory muscles were noted in Jandt et al.^[19]^ Twenty-one stroke survivors with hemiparesis were evaluated according to maximal inspiratory pressure (MIP) and maximal expiratory pressure (MEP) through the mouth, spirometry (FVC, FEV1, FEV1/FVC%, PEF), and the TIS. A positive correlation between the TIS and PEF values (r = 0.49, *P* = .02) and between the TIS and MEP values (r = 0.52, *P* = .02) was observed.

It should be emphasized that in recent years, there has been an increasing interest in the assessment of the PNF method in people with stroke, not only related to the respiratory system, which justifies the need and importance of this research. Research of various research teams shows the beneficial effect of this method on the activity of additional inspiratory muscles,^[28]^ gait parameters,^[29]^ mobility,^[30]^ balance,^[30–33]^ muscle stiffness,^[34]^ daily living, and quality of life.^[35]^

*Limitation*: The evaluation was conducted after the single-session intervention, but not after a series of respiratory therapy treatments, therefore, this research should be considered as a report include only immediate effects of intervention Also, it seems that there is a need for evaluation in both the chronic and acute phases of stroke, and the effects of stimulations would have to be compared with other types of breathing exercises. The limitation of this study is the lack of a long-term assessment. In continued studies by our team, such evaluation has been included.

Respiratory stimulation through PNF contributed to an improvement of the FEV1/FVC% parameter in long-term ischemic stroke survivors. So far, no research has been conducted to provide scientific proof of the superiority of using specific kinesitherapeutic methods in stroke survivors or to assess the influence of therapeutic interventions on ventilation. This study highlights the importance of therapeutic action directed towards proper mechanics of ventilation, even long time after the stroke episode.

## 5 Conclusions

A single application of respiratory stimulation through PNF positively affect air flow in the respiratory tract. Application of PNF stimulation contributed to an increase in the FEV1/FVC% parameter. However, no positive changes were noted in the other parameters, which would provide proof of the beneficial effect of facilitation on the respiratory system function. More research is necessary to assess if therapy involving a series of respiratory interventions through PNF improves respiratory parameters more than a single stimulation.

## Author contributions

**Conceptualization:** Lucyna Ptaszkowska, Kuba Ptaszkowski, Tomasz Halski, Jakub Taradaj, Robert Dymarek, Malgorzata Paprocka-Borowicz.

**Data curation:** Lucyna Ptaszkowska, Kuba Ptaszkowski.

**Formal analysis:** Lucyna Ptaszkowska, Kuba Ptaszkowski.

**Funding acquisition:** Kuba Ptaszkowski.

**Investigation:** Lucyna Ptaszkowska, Tomasz Halski, Malgorzata Paprocka-Borowicz.

**Methodology:** Lucyna Ptaszkowska, Kuba Ptaszkowski, Tomasz Halski, Jakub Taradaj, Robert Dymarek, Malgorzata Paprocka-Borowicz.

**Project administration:** Lucyna Ptaszkowska.

**Supervision:** Lucyna Ptaszkowska, Tomasz Halski, Malgorzata Paprocka-Borowicz.

**Validation:** Lucyna Ptaszkowska, Kuba Ptaszkowski, Jakub Taradaj, Malgorzata Paprocka-Borowicz.

**Writing – original draft:** Lucyna Ptaszkowska, Malgorzata Paprocka-Borowicz.

**Writing – review & editing:** Lucyna Ptaszkowska, Kuba Ptaszkowski, Tomasz Halski, Jakub Taradaj, Robert Dymarek, Malgorzata Paprocka-Borowicz.
